# Influence of Phosphorylation and Acetylation on Structural, Physicochemical and Functional Properties of Chestnut Starch

**DOI:** 10.3390/polym14010172

**Published:** 2022-01-02

**Authors:** Chang Liu, Hejing Yan, Suwen Liu, Xuedong Chang

**Affiliations:** 1 College of Food Science and Technology, Hebei Normal University of Science and Technology, Qinhuangdao 066000, China; 3530@hevttc.edu.cn (H.Y.); lsw3318@hevttc.edu.cn (S.L.); cxd0683@hevttc.edu.cn (X.C.); 2 Collaborative Innovation Center of Hebei Chestnut Industry, Qinhuangdao 066000, China; 3 Hebei Key Laboratory of Active Components and Functions in Natural Products, Qinhuangdao 066000, China

**Keywords:** chestnut starch, chemical modifications, phosphorylation, acetylation, physicochemical properties

## Abstract

Chestnut is popular worldwide for its unique flavor, high eating quality and nutrition. Here, we evaluated the influence of phosphorylation and acetylation on the structural, physicochemical and functional properties of chestnut starch. Scanning electron micrographs showed the agglomeration of starch granules and the appearance of numerous dents on the starch granule surface under phosphorylation and acetylation. X-ray diffractograms confirmed that the modification treatments did not affect the C-type crystal pattern, but reduced the relative crystallinity of the chestnut starch, particularly phosphorylation. Moreover, modification improved the paste transparency of the starch. Differential scanning calorimeter analysis revealed that the gelatinization temperature and enthalpy of the starch decreased with the increasing substitution degree, particularly in phosphorylated starch. The Rapid Visco Analyser analysis demonstrated that phosphorylation could greatly improve the pasting properties of chestnut starch. In addition, phosphorylated and acetylated starch had a smaller amount of slowly digested starch and a larger amount of resistant starch relative to native chestnut starch. In conclusion, the functional and physicochemical properties of chestnut starch can be significantly improved through phosphorylation and acetylation, demonstrating its great application potential as a food additive.

## 1. Introduction

Starch is the main carbohydrate reserve for many higher plants as well as the most abundant natural and renewable biopolymer next to cellulose. It has diverse applications in both food and industrial production. However, the application of native starch at the industrial level has been largely hindered by its low flowability and shear stress resistance, thermal decomposition during processing and high syneresis and retrogradation [1,2]. For various purposes in industrial applications, different methods have been used to modify starch, which can be generally classified into chemical, physical and enzymatic modifications [3]. The chemical modification of starch, which introduces functional groups at the molecular level to alter the bulk properties, can improve the inherent properties of starch, including its digestibility, solubility, thickening power, pasting properties and shear stability [4]. The repeating glucose units in starch molecules can provide abundant hydroxyl groups (at positions of C2, C3 and C6), which are excellent sites for the incorporation of functional groups [5]. The properties achieved by the chemical modification of starch are influenced by various factors, such as the botanical source, reaction conditions (reactant concentration, reaction time, pH and the presence of a catalyst), the type of substituent, degree of substitution and substituent distribution in starch molecules [6].

Phosphorylated starch is an abundantly produced and widely applied starch with chemical modifications. The introduction of phosphate groups into starch chains leads to repulsion between the phosphate groups on adjacent chains and elevates hydration. Therefore, increasing the phosphorylation degree can alter the physicochemical properties of starch, even when the degree of substitution (DS) is very low [7,8]. Nevertheless, the effect of this modification is highly dependent on the source of starch and the reaction conditions. It has been reported that phosphorylation can improve the solubility, swelling ability, retrogradation, freeze–thaw stability and solution transparency of starch [9,10].

Acetylation is another approach for starch modification through the substitution of hydroxyl groups of glucose with acetyl groups [11]. The introduction of acetyl groups will disrupt the originally ordered structure of native starch as well as decrease the electrostatic interaction of amylose and amylopectin chains. Acetylated starch has different functions depending on the DS. At a lower DS (0.01–0.2), acetylated starch is characterized by its various desirable properties, including high thickening power, low gelatinization temperature, low-temperature stability, its improvement of the clarity of cooked food and reduced tendency of retrogradation [12]. Previous studies have indicated that acetylation can improve the water absorption and solubility of starch and delay starch retrogradation [13,14]. The reaction conditions can also greatly influence the functional properties of the starch derived from different botanical origins [15].

The chestnut (*Castanea mollissima* Bl.), which belongs to the Fagaceae family, is a traditional nut and also a popular food around the world [16,17]. Chestnuts are widely distributed and consumed in Asia, America and Europe, and the chestnut yield has been increasing over the last few decades. China is as one of the top producers of chestnuts [18,19]. The chestnut also has a long history of being used as a traditional Chinese medicine. As one of the most important components in a chestnut, starch accounts for 50–80% of the dry matter [20,21]. Besides starch, chestnuts also contain some important functional components, such as minerals, polyphenols, vitamins and dietary fibers [22]. There have been numerous studies of chestnuts as a new source of starch. Liu et al. reported that chestnut starch has unique physicochemical properties, such as high swelling power, freeze–thaw stability and pasting viscosity and a low gelatinization temperature [20]. Moreover, Liu et al. analyzed the structural and physicochemical properties of large and small granules of Chinese chestnuts and evaluated the effect of particle size on starch properties [23]. Varieties and geographical distribution were found to significantly influence the characteristics of chestnut starch [21]. In addition, some studies have reported the effects of drying methods and phosphorylation on the structural and functional properties of chestnut starch [24,25]. With the rapid development of starch-based food, chestnut starch shows increasing application potential.

To date, the physicochemical properties of modified starch from potato, corn, rice and pea have been extensively studied. However, little research attention has been paid to the properties of acetylated and phosphorylated starch from chestnuts. Considering the promising opportunities that modification may bring to starch production, we investigated the effects of different degrees of acetylation and phosphorylation on the structure and functionality of chestnut starch from the perspectives of surface morphology, crystallinity, swelling power, paste clarity, thermal properties, pasting properties and in vitro digestibility of chestnut starch. Our findings provide insights into the modification methods that may help to improve the functional and physicochemical properties of chestnut starch for its better application in food and other industries.

## 2. Materials and Methods

### 2.1. Materials

The fruit of the Chinese chestnut variety (Yanshan Zaofeng), one of the chestnut varieties with the largest yield and the best flavor in the Yanshan region, were collected from the Chestnut Industry Collaborative Innovation Center of Hebei province, China, in the 2020 season. The fruit were stored at 4 °C before analysis. Chestnut starch was obtained through the alkaline method following the procedures of our previous report [20]. The chestnut starch had a high purity of >98%, with 0.13% ash, 0.23% protein and 0.80% lipid.

A glucose oxidase/peroxidase (GOPOD) kit and amyloglucosidase (E-AMGDF, 3300 U/mL) were provided by the Megazyme International Ireland Ltd. (Bray Co., Wicklow, Ireland). Amylose (A0512) and amylopectin (A8515) from potato starch were purchased from Sigma Chemical Co. (St. Louis, MO, USA).

### 2.2. Chemical Modification of Starch

#### 2.2.1. Starch Phosphorylation

Phosphorylated chestnut starch was prepared according to Hu et al. with minor modifications [25]. Briefly, sodium dihydrogen phosphate dihydrate (NaH_2_PO_4_·2H_2_O) and urea were mixed at a ratio of 10:1 (*w*/*w*). The mixture was then dissolved in distilled water for preparing a 7% (*w*/*v*) NaH_2_PO_4_ salt solution for phosphorylation. Chestnut starch (40 g, dry basis) was first suspended in an 80 mL NaH_2_PO_4_ solution, followed by gradual adjusting of the pH to 6.0 with 0.1 M solution of NaOH. The mixture was stirred for 30 min and then vacuum filtered. The starch was dried in an oven at 40 °C for 24 h, and then heated at 145 °C for 60, 120 or 180 min to obtain phosphorylated starch with different DS. After heat treatment, the resultant retentate was washed several times with distilled water to removed residual phosphorus salts. Finally, the sample was dried in an oven at 30 °C, ground and then sieved (80 μm mesh).

#### 2.2.2. Starch Acetylation

Chestnut starch acetylation was performed according to the method of Elijah et al. with some modifications [1]. Briefly, native chestnut starch (40 g, dry basis) was dispersed in 60 mL of distilled water and stirred for 20 min at 40 °C to obtain a 40% (*w*/*v*) starch suspension. The pH of the suspension was adjusted to 9.0 by using a 3.0% (*w*/*v*) solution of NaOH. Acetic anhydride (6 g) was added dropwise to the stirred slurry, with the pH being maintained at 8.8–9.0 with the 3.0% NaOH solution. Then, the reaction was conducted at 40 °C for 30, 60 and 90 min. The pH of each starch slurry sample was adjusted to 6.5 by using 2.0% (*w*/*v*) HCl, and then the sample was centrifuged at 6000× *g* for 20 min. The obtained residue was washed repeatedly with distilled water until it had a constant pH of 6.5, and then oven dried at 30 °C for 48 h. Finally, the dried starch was ground and sieved (80 μm mesh), and acetylated starch was obtained.

The starches modified with phosphorylation or acetylation are listed in Table 1, which were named as PCS-1, PCS-2 and PCS-3 and ACS-1, ACS-2 and ACS-3, respectively.

### 2.3. Determination of Substitution Degree

#### 2.3.1. Phosphorylated Starch

The phosphorus content in the phosphorylated chestnut starch was measured using the ammonium molybdate and vanadate spectrophotometric method [7]. The DS was calculated using the following formula:

$$\mathrm{DS}=\;\frac{162\times p}{3100-102\times p}$$

where *p* indicates the % phosphorus content (*w*/*w*, dry basis).

#### 2.3.2. Acetylated Starch

The DS was determined according to Shubeena et al. with some modifications [26]. A blank sample (unacetylated starch) was also used as a blank control. Percent acetylation was calculated with the following formula:

$$\mathrm{Acety}\%=\frac{\left(\mathrm{Blank}-\mathrm{Sample}\right)\times \mathrm{Molarity}\;\mathrm{of}\;\mathrm{HCL}\times 0.043\times 100}{\mathrm{Weight}\;\mathrm{of}\;\mathrm{Sample}\;\left(g\right)}$$

where blank and sample stand for the titration volumes of the blank and sample (in mL), respectively. The DS was calculated as the average sites with substituent groups per glucose as follows:

$$\mathrm{DS}=\frac{\left(162\times \mathrm{Acetyl}\%\right)}{4300-\left(42\times \mathrm{Acety}\%\right)}$$

### 2.4. Determination of the Apparent Amylose Content

Apparent amylose content in the starch was measured using the iodine binding method with a calibration curve of zero to 40% amylose mixed with amylopectin [27].

### 2.5. Morphological Properties

The images of the chestnut starch were taken using the SU-8010 Scanning Electron Microscope (Hitachi Company, Tokyo, Japan). The accelerating voltage was 5 kV for imaging. Different samples were photographed under SEM at a magnification of 4000× and 6000×.

### 2.6. X-ray Diffraction (XRD)

X-ray diffraction analysis was performed with a D/max-2500vk/pc X-ray diffractometer (Rigaku Corporation, Tokyo, Japan). The equilibration of the starch was performed with a saturated potassium chloride (KCl) solution at room temperature for one week before analysis [20]. The XRD pattern was determined from 3° to 40° (2θ) with a scanning speed of 1°/min and a step size of 0.02°. The Origin software was used to calculate the relative crystallinity as the ratio of the crystalline area to the total area (v9.0, Microcal Inc., Northampton, MA, USA).

### 2.7. Fourier Transformed Infrared (FTIR) Spectroscopy

FTIR spectroscopy was performed for all samples using a Tensor 27 FTIR Spectrometer (Bruker Corporation, Berlin, Germany) over a range between 4000 and 400 cm^−1^. Starch samples were diluted with KBr (starch/KBr, 1:100, *w*/*w*) and pressed into tablets before measurement. The background value from pure KBr was acquired before scanning of the sample.

### 2.8. Determination of Starch Swelling Power

The starch swelling power was determined in excess water at temperature from 40 °C to 90 °C following the descriptions of Konik Rose et al. [28].

### 2.9. Measurement of Starch Paste Clarity

The clarity of the chestnut starch gel was measured as described in our previous report [29]. The aqueous suspension of starch (1%, *w*/*v*) was prepared and heated in a water bath at 90 °C for 30 min with constant stirring. The paste clarity was determined as the percentage transmittance at 650 nm.

### 2.10. Determination of Thermal Properties

The thermal properties of the starch were measured using a differential scanning calorimeter (DSC, 200F3, Netzsch, Selb, Germany) equipped with a thermal analysis data station. Starch (3 mg, dry basis) and 15 μL of H_2_O were mixed and sealed in an aluminum pan and allowed to stand still for 1 h before analysis. The samples were heated from 20 °C to 100 °C at a rate of 10 °C/min. The onset (T_o_), peak (T_p_) and conclusion (T_c_) of the gelatinization temperature and gelatinization enthalpy (ΔH) were recorded.

### 2.11. Determination of Pasting Properties

The pasting profile of the starch was monitored using a Rapid Visco Analyser (RVA-TECMASTER, Perten Instruments Ltd., Sydney, NSW, Australia). Starch (3.0 g, dry weight) was first dispersed in distilled water (25 mL), held at 50 °C for 1 min, heated to 95 °C (12 °C/min), held for 3.3 min, and then cooled (12 °C /min) to 50 °C and held for 2 min. The mixing paddle was set at a speed of 960 rpm for the first 10 s, and then at 160 rpm in the rest of the testing period.

### 2.12. In Vitro Starch Digestibility

In vitro starch digestibility was determined by a procedure described in our previous report [20], which was modified from the Englyst procedure [30]. At specified time points, the glucose concentration in the digestion solution was determined with the Megazyme GOPOD kit. Starches were classified according to the hydrolysis rate: rapidly digested starch (RDS; digested within 20 min), slowly digested starch (SDS; digested between 20 and 120 min) and resistant starch (RS; undigested after 120 min). The RS content as a percentage was calculated as 100 − (RDS + SDS).

### 2.13. Statistical Analysis

The results are presented as mean value ± standard deviation of at least triplicates, except for XRD, which was performed only once. A one-way analysis of variance (ANOVA) followed by a Duncan test (*p* < 0.05) was used to examine significant differences among experimental mean values using the SPSS 19.0 Statistical Software Program (SPSS Inc., Chicago, IL, USA).

## 3. Results and Discussion

### 3.1. Degree of Substitution 

The level of starch modification can be indicated by the amount of substitute introduced during the chemical modification process. At the reaction times of 60, 120 and 180 min, the DS of phosphorylated starch was 0.030, 0.071 and 0.095, respectively. The phosphorus content was lower than 0.183% in all phosphorylated samples, which was below the maximum limit allowed for food application purposes according to the Food Chemical Codex (0.4%) [31]. The DS of the modified samples significantly increased with the increasing reaction time, which is consistent with the results of Yang et al. [9]. Phosphorylation for 180 min resulted in a higher DS of the starch than phosphorylation for 60 and 120 min, probably because of the substitution of more hydrogens by phosphoric acid groups on the hydroxyl groups in each glucosyl unit with the increasing reaction time under the high temperature of 145 °C. The DS values obtained in the present study were close to those previously reported for chestnut starch under similar phosphorylation conditions [25].

At the reaction times of 30, 60 and 90 min, the DS of the acetylated starch was 0.010, 0.020 and 0.024, respectively. Similarly, the starch acetylated for 90 min showed a higher DS than that acetylated for 30 and 60 min. The acetyl content ranged from 0.18 to 0.38% for the different reaction times, which is also below the maximum limit (2.5 g/100 g) for acetylated starch recommended by the United States Food and Drug Administration for food applications [32,33]. Generally, low-DS acetylated starch is produced by esterifying native starch with acetic anhydride under the action of alkaline catalysts [5]. Compared with the corn, potato and cassava starches treated with acetic anhydride, the acetylated chestnut starch generated under similar conditions in this study had slightly lower DS values [14,34,35], which may be attributed to different starch source, properties and experimental conditions.

### 3.2. Apparent Amylose Content

Table 1 presents the influence of modification treatments on the apparent amylose content in chestnut starch. The amylose contents of chemically modified starch varied between 9.55% and 20.86%, and the values were significantly lower than that of native starch. Phosphorylation and acetylation were found to have similar effects on the content of amylose in starch in previous research [36]. In this study, acetylation resulted in a higher amylose content in chestnut starch than phosphorylation. The introduction of phosphate groups into starch chains can destroy the helical structure of amylose, resulting in a lower apparent amylose content. It has been reported that the presence of acetyl groups can hinder the formation of the helical structure in amylose due to the effect of sterical hindrance and thereafter reduce the formation of amylose–iodine complex. Lawal et al. demonstrated that acetylation decreased the amylose content in corn, cassava and sweet potato starch, which was attributed to the leaching of amylose during acetylation [37]. On the contrary, some reports showed that acetylation slightly increased the amylose content in pigeon pea, chickpea and sorghum starch relative to native starch [38,39,40]. These differences can be attributed to the different plant sources of starch or different methods of amylose quantification.

### 3.3. Morphology of Starch Granules

**Figure 1:**
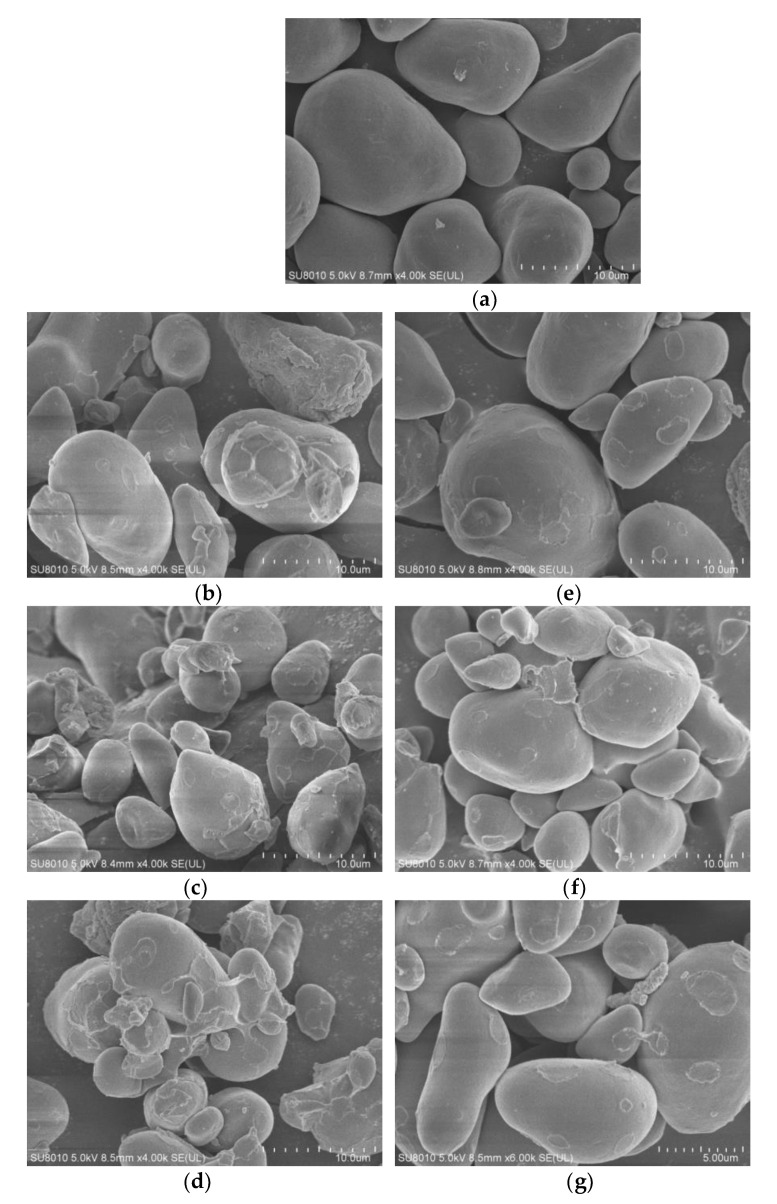
Scanning electron micrographs (SEM) of native, phosphorylated and acetylated chestnut starch (4000×, 6000×): (**a**) NS; (**b**) PCS-1; (**c**) PCS-2; (**d**) PCS-1; (**e**) ACS-1; (**f**) ACS-2; (**g**) ACS-3.

The SEM images of native and modified starches are presented in Figure 1. The native starch granules exhibited diverse morphologies, including round, irregular and triangular shapes (Figure 1a). In native chestnut starch, there were both large and small granules with smooth surfaces and no cracks or pores. However, the granules of phosphorylated starch exhibited more dents and pores on the surface with increasing reaction times, and phosphorylation led to a noticeable aggregation of starch granules (Figure 1b–d). Native starch underwent apparent damage after phosphorylation. With the increase in DS, some granules of phosphorylated starch were observed to show evident decreases in structural integrity, suggesting the disruption of the ordered crystalline structure of starch due to the breaking of hydrogen bonds. These results are in line with the findings in previous studies [41,42].

Acetylation led to the appearance of some concave pits on the starch granule surfaces and a slight aggregation of the starch granules, but the chestnut starch granules still maintained a relatively complete structure (Figure 1e–g). The starch granule aggregation after acetylation can be ascribed to the incorporation of acetyl groups into the starch molecules. The rough surface or aggregation of the starch granules may also be attributed to surface gelatinization due to the addition of NaOH when the pH was kept at 9.0 during acetic anhydride addition. In previous reports, similar results were obtained for the morphology of acetylated corn, potato and barley starch [13,38,42]. However, these findings are not in conformity with the observation that acetylation led to little change in the granular morphology of rice, amaranth and buckwheat starch [43,44,45]. Chestnut starch granules are fragile in nature and thus may be more significantly affected by chemical modification than other starches.

### 3.4. X-ray Diffractions Analysis

**Figure 2:**
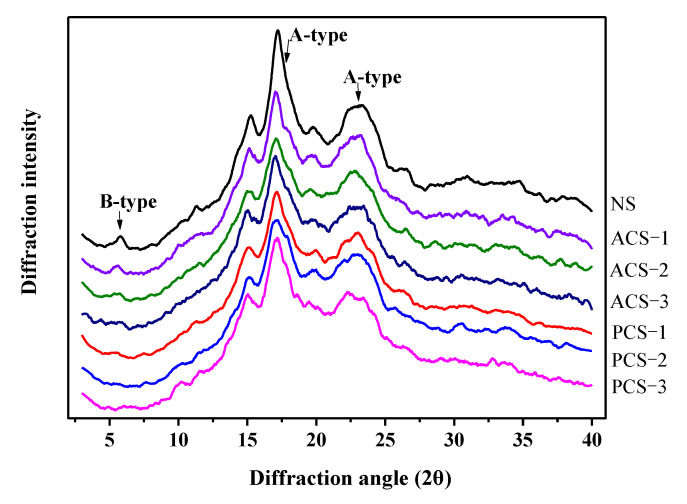
XRD spectra of native, phosphorylated and acetylated chestnut starch.

Figure 2 presents the XRD spectra for modified and native chestnut starch. For native chestnut starch, a characteristic C-type X-ray diffraction pattern was observed, and there were diffraction peaks at 5.6°, 15.4°, 17.0°, 20.0° and 23.0° (2θ). The modification did not alter the crystalline type of chestnut starch, which exhibited a characteristic C-type crystallinity pattern. After modification, the intensity of some characteristic diffraction peaks slightly diminished due to the substitution of some hydroxyl groups by phosphate or acetyl groups in the amorphous and crystalline area during modification. The peak intensity at 5.6° (2θ) decreased after phosphorylation, indicating a decrease in the number of “B-type” polymorphs in phosphorylated starch, presumably due to the damage of the long-range order of double helices.

The decrease in peak intensity after modification compared with that of native starch indicated that modification altered the relative crystallinity of chestnut starch. The relative crystallinity of starch followed the order of native starch > acetylated starch > phosphorylated starch. The increase in the DS of the modified starch was generally accompanied by decreases in relative crystallinity. Chemical modification can reduce the hydroxyl groups that may participate in the generation of intra/inter-molecular hydrogen bonds necessary for helical crystalline structure formation in starch. In the present study, phosphorylation decreased the formation of intra/inter-molecular hydrogen bonds more significantly than acetylation, making the crystalline structure of starch granules less ordered. Acetic anhydride shows a poor penetrating ability in starch granules. Acetylation occurs in all amorphous regions as well as at the outer lamellae of the crystalline regions, rather than throughout the crystalline regions of the whole starch granule. The decreasing degree of the crystalline order in starch after acetylation is dependent on the reaction time and DS as reported by Sindhu et al. [45].

### 3.5. Fourier Transform Infrared (FTIR) Spectroscopy

**Figure 3:**
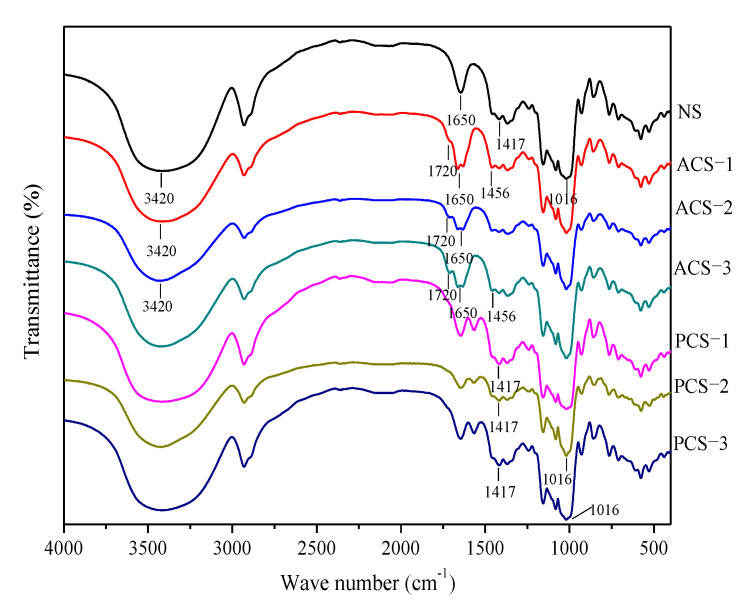
FTIR spectra of native, phosphorylated and acetylated chestnut starch.

Figure 3 shows the FTIR spectra of native and modified chestnut starch. The chemically modified starch exhibited changes in the intensity of some bands accompanied by the appearance of some new bands. In the region of 3000–3700 cm^−1^, there was a decrease in the intensity of the band, indicating a decline in the stretching vibration of O-H bonds, which were attributed to the substitution of hydroxyl groups by phosphate groups and acetyl groups in phosphorylated and acetylated starch. The spectra of the phosphorylated starch showed a characteristic absorption peak at 1016 cm^−1^ relative to that of native starch, which was related to the antisymmetric stretching vibration of the P-O-C bond. A new band appeared at 1417 cm^−1^, which has been reported to be the characteristic of the P = O stretching vibration [10].

Relative to those of native starch, the FTIR spectra of acetylated starch with a different DS exhibited a new band at 1720 cm^−1^, which originates from the stretching vibration of C = O carbonyl ester and proves the occurrence of acetylation [1,38]. A characteristic absorption band observed at 1456 cm^−1^ was assigned to the CH_3_ antisymmetric deformation vibration, which also confirms the increment of acetyl groups in acetylated starch molecules. Furthermore, there was a decrease in the intensity of the band at 1650 cm^−1^ in acetylated starch because of the incorporation of acetyl groups into the starch, which is due to the lower affinity of acetylated starch to water. Luo and Shi also reported a hydrophobic character of acetylated maize starch [46]. Similar findings have been reported for acetylated maize, bean and amaranth starch [45,47].

The absorbance bands at 1047 cm^−1^ and 1022 cm^−1^ have been reported to be sensitive to the ordered structure and the amorphous structure in starch, respectively. Hence, the ratio of absorbance at 1047 cm^−1^ and 1022 cm^−1^ was employed for characterizing the short-range molecular order of double helices [24]. As a result, the ratio of the modified starch decreased gradually with the increasing DS (Table 1). Moreover, the ratio was the highest in native chestnut starch (1.574), and that in acetylated and phosphorylated chestnut starch with a different DS ranged from 1.512 to 1.292 and from 1.334 to 1.135, respectively. Combining the FTIR and XRD results, it can be concluded that phosphorylation can more significantly disrupt the short-range and long-range molecular order of starch than acetylation.

### 3.6. Paste Clarity

**Figure 4:**
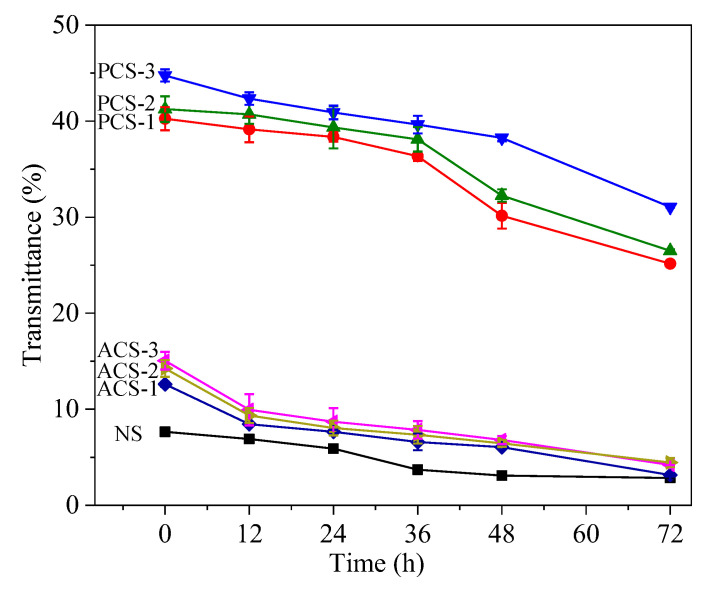
Light transmittance of native, phosphorylated and acetylated chestnut starch.

Starch paste clarity is a significant quality parameter closely associated with the botanical source of starch. Figure 4 presents the effect of storage and modification on the clarity of chestnut starch paste. The transmittance (%) of native and modified starch decreased progressively with the extension of storage. Chemical modification enhanced the paste clarity of chestnut starch, particularly phosphorylation. This result suggests that the charged phosphate groups can help to keep the hydration of starch molecules and reduce their recrystallization degree. The transmittance of phosphorylated starch increased with the DS, as phosphate groups could strengthen the bonds between starch and water molecules, which is consistent with previous findings [25].

Acetylation can also improve the clarity of chestnut starch, possibly because the introduction of acetyl groups hinders the formation of an ordered structure after gelatinization, and therefore suppresses retrogradation to result in a more fluid paste with higher clarity. Similar effects of acetylation on paste clarity have been reported for various types of starch [14,48]. Higher paste clarity is a desired property for the production of some foods, such as salad dressings and confectionery products.

### 3.7. Swelling Power

Clearly, the swelling power of chestnut starch phosphorylated and acetylated at 40 °C to 90 °C with a different DS was in the range of 12.30–24.89 g/g and 1.32–15.75 g/g, respectively (Table 2). Native and acetylated starch had no swelling power at room temperature due to the bonding force between starch molecules, whereas phosphorylated starch granules could swell at 40 °C, because the introduction of negatively charged phosphate groups disrupts the hydrogen bonding between starch molecules to facilitate water penetration and swelling. A higher DS leads to the incorporation of more hydrophilic groups into the starch, which would enhance the hydrophilicity of phosphorylated starch and further increase water penetration and starch swelling power.

Acetylated chestnut starch showed a similar swelling power to native starch, which might be due to the low DS of acetylation. Moreover, the FTIR results implied that the starch with the incorporation of acetyl groups shows lower water affinity. Similar results were reported for rice starch [11]. However, these findings are not in agreement with those of some other research, which stated that acetylation treatment increases the swelling power of starch [6,14]. Although starches from various sources generally have similar structures, they may differ greatly in microstructure and ultrastructure, which have potential effects on the chemical modification process.

### 3.8. Thermal Properties of Starch

**Figure 5:**
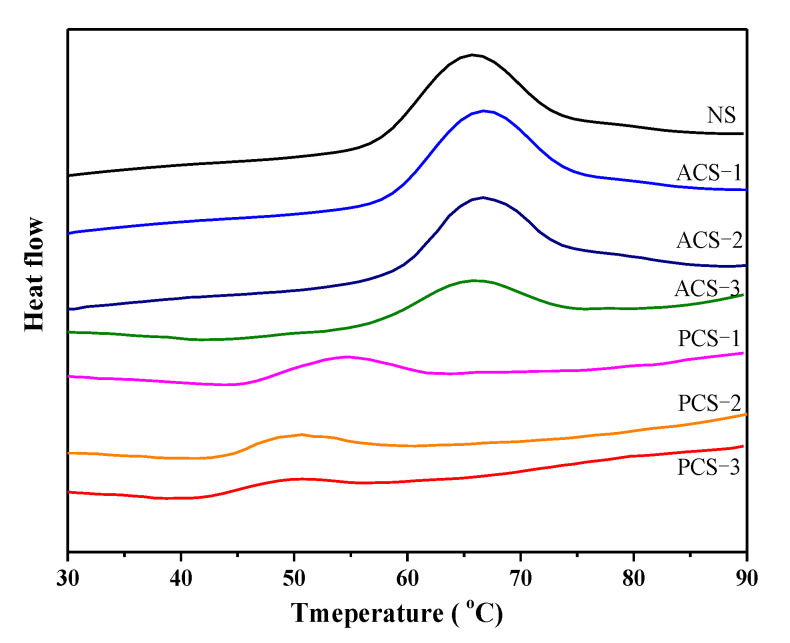
DSC thermograms of native, phosphorylated and acetylated chestnut starch.

Figure 5 and Table 3 show the DSC thermograms and the corresponding thermal transition parameters of the native and modified chestnut starch. Phosphorylation significantly decreased both the gelatinization temperature (T_o_, T_p_ and T_c_) and gelatinization enthalpy (ΔH) of the chestnut starch with the increase in the DS, indicating the accelerated destabilization of the starch structure, which requires less energy for gelatinization. The modification process, particularly phosphorylation, causes the collapse of the starch structure, which will facilitate internal molecular disorganization in the process of gelatinization. The decline in the gelatinization temperature is possibly due to the variations in starch granules, such as the early uncoiling of amylopectin double helices during phosphorylation, which is similar to the results reported in a previous study [9]. However, these results are not in conformity with the finding that phosphorylation increases the gelatinization temperature of Taro starch [2]. The changes in thermal properties of starch are affected by various factors, such as granule rigidity, the presence or absence of lipids, the DS and the amylose to amylopectin ratio.

As shown in Table 3, acetylated starch (ACS-2 and ACS-3) showed a decrease in ΔH compared with native starch, and a higher DS would lead to a more significant decrease in ΔH. Acetylated starch had a higher gelatinization temperature and ΔH than phosphorylated starch, indicating that phosphorylation can more significantly disrupt the crystalline structure and double helix in starch granules than acetylation. The insertion of acetyl groups into starch chains would disturb the granular structure and subsequently cause a slight decrease in the gelatinization temperature (T_o_ and T_p_). The reduction of ΔH may be attributed to the disruption of double helices in both crystalline and non-crystalline regions of starch granules during acetylation, which can be confirmed by the XRD and FTIR results. Similar trends for the thermal parameters have been reported for acetylated starch from different sources [14,37,45]. The reduction of the gelatinization temperature and gelatinization enthalpy can help to decrease the energy cost in food processing.

### 3.9. Pasting Properties

Table 4 presents the pasting characteristics of the native and modified starch. Phosphorylated starch (50.2 °C) and native starch (74.2 °C) had the lowest and highest pasting temperature, respectively. The phosphorylated starch had the highest values of peak, trough and breakdown viscosities. In addition, the phosphorylated starch showed increases in viscosity with increasing DS. In phosphorylated starch, the presence of hydrophilic phosphate groups can facilitate water percolation within the amorphous regions of granules, resulting in a higher peak viscosity [31]. Phosphorylated chestnut starch with a higher DS showed a higher viscosity, which is more suitable for end products with higher viscosity and lower retrogradation and pasting temperature.

According to the RVA results, the acetylation process resulted in a slight decrease in the pasting temperature (from 74.2 °C to 71.9 °C), and did not significantly change the peak, trough and final viscosities, but decreased the breakdown and setback viscosity relative to those of native starch. The presence of acetyl groups hinders the formation of an ordered structure, thus delaying starch retrogradation. These results are inconsistent with the finding of some studies that acetylation may increase the viscosity of starch [16,26,34]. In addition to the starch source, the pasting viscosity may also be related to the granular structure and experimental conditions.

Moreover, these properties may also be influenced by the size of the substituents. The phosphate group has a larger size than the acetyl group. The introduction of larger substituting groups to amylose or the outer branches of amylopectin starch can create more free space between starch macromolecules, which will hinder the establishment of intermolecular or intramolecular associations between the individual chain segments due to the effect of steric hindrance.

### 3.10. In Vitro Digestion

Table 5 shows the digestibility of modified and native chestnut starch. RDS, SDS and RS accounted for 8.2%, 33.6% and 58.2% of the total weight in native chestnut starch, respectively. Native starch granules are indigestible because the starch molecules are arranged in a relatively dehydrated state, which limits their contact with hydrolytic enzymes. However, gelatinization can disrupt this complex structure, making the starch molecules accessible to digestive enzymes [49]. Phosphorylation and acetylation significantly increased the proportion of RDS and RS while decreasing that of SDS. The increase in RDS indicated the destruction of the integrity of the granules and the degradation of the ordered chain structure during modification, which can increase the susceptibility of starch to enzymatic attack. The chemically modified starch showed significant decreases in SDS relative to native starch, which was as low as 4.6–13.2%.

Interestingly, the modified starch showed a higher proportion of RS than native starch in this study, and acetylated starch had a relatively higher RS proportion (81.4–84.0%) than phosphorylated starch (62.7–76.2%). These results may be explained by the introduction of phosphate and acetyl groups to the starch molecules, which may retard the formation of the enzyme-substrate complex. Some factors that may affect starch digestibility include the starch source, the amylose to amylopectin ratio, crystallinity, the granule size and the amylopectin molecular structure. The main reason for the higher RS content in chemically modified starches is the high specificity of digestive enzymes that do not recognize the chemically modified structure of amylose and amylopectin chains, which results in the high values of nonhydrolyzed starch fractions. The results of the digestibility test implied that a high RS can be achieved through modification, especially acetylation. RS can be added to some foods to reduce caloric content and the postprandial insulinemic response after consumption.

## 4. Conclusions

Phosphorylation and acetylation were found to significantly influence the physico-functional and chemical properties of chestnut starch in this work. An increase in the reaction time can increase the DS of the modified starch. Phosphorylation and acetylation led to the appearance of dents on the surface and the aggregation of starch granules. Chemical modification does not influence the C-type crystalline pattern of chestnut starch but reduces the relative crystallinity, indicating that the crystalline structure is disrupted. Chemical modifications, especially phosphorylation, disrupt the long- and short-range molecular order of double helices in chestnut starch, which further decreases the gelatinization temperature and gelatinization enthalpy of the starch. Relative to native chestnut starch, phosphorylated chestnut starch has a higher swelling power, lower pasting and gelatinization temperature, higher paste clarity and better viscosity characteristics with increasing DS. Acetylated starch has a higher proportion of RS than native starch, suggesting a decrease in digestibility. Overall, our findings demonstrate that different technologies for starch modification may be employed to generate different characteristics in the final products, which will greatly improve the applicability of chestnut starch in the food industry.

## Figures and Tables

**Table 1:** Degree of substitution, apparent amylose content, relative crystallinity and FTIR absorbance ratio at 1047 and 1022 cm^−1^ of phosphorylated and acetylated chestnut starch.

Treatment	Reaction Time (min)	Apparent Amylose (%)	Relative Crystallinity (%)	1047/1022 cm^−1^
Native starch	-	25.06 ± 0.12a	24.22 ± 0.02a	1.574 ± 0.021a
Phosphorylated starch	60	14.83 ± 0.12d	19.82 ± 0.1b	1.334 ± 0.046d
	120	12.22 ± 0.06e	18.95 ± 0.3b	1.267 ± 0.028e
	180	9.55 ± 0.04f	16.20 ± 0.2c	1.135 ± 0.062f
Acetylated starch	30	20.86 ± 0.11b	22.73 ± 0.2d	1.512 ± 0.016b
	60	18.56 ± 0.11c	22.56 ± 0.3b	1.432 ± 0.074c
	90	18.93 ± 0.05c	20.66 ± 0.1e	1.292 ± 0.035e

Values are means ± SD. Values with the same letters in the same column are not significantly different (*p* < 0.05). NS: native chestnut starch; PCS-1: chestnut starch phosphorylated for 60 min; PCS-2: chestnut starch phosphorylated for 120 min; PCS-3: chestnut starch phosphorylated for 180 min; ACS-1: chestnut starch acetylated for 30 min; ACS-2: chestnut starch acetylated for 60 min; ACS-3: chestnut starch acetylated for 90 min.

**Table 2:** Swelling power (g/g) of native, phosphorylated and acetylated chestnut starch.

Samples	40 °C	50 °C	60 °C	70 °C	80 °C	90 °C
NS	1.88 ± 0.03d	2.10 ± 0.16c	3.14 ± 0.05b	9.59 ± 0.01b	14.50 ± 0.17b	15.16 ± 0.18b
PCS-1	12.30 ± 0.51c	18.04 ± 0.10b	20.21 ± 0.18a	22.45 ± 0.87a	23.35 ± 0.37a	24.10 ± 0.25a
PCS-2	16.57 ± 0.11b	22.48 ± 0.28a	20.21 ± 0.61a	22.71 ± 0.90a	23.78 ± 0.54a	24.64 ± 0.16a
PCS-3	19.35 ± 0.46a	22.11 ± 0.37a	22.51 ± 0.34a	24.34 ± 0.15a	23.90 ± 0.58a	24.89 ± 0.17a
ACS-1	1.80 ± 0.04d	2.22 ± 0.03c	3.70 ± 0.12b	9.07 ± 0.20b	13.41 ± 0.36b	15.75 ± 0.33b
ACS-2	1.32 ± 0.02d	2.11 ± 0.08c	3.70 ± 0.06b	8.41 ± 2.72b	13.21 ± 0.43b	14.96 ± 0.58b
ACS-3	1.75 ± 0.03d	2.44 ± 0.19c	3.65 ± 0.19b	9.39 ± 0.29b	13.92 ± 0.28b	14.73 ± 0.42b

Values are means ± SD. Values with the same letters in the same column are not significantly different (*p* < 0.05). Note: abbreviations are the same as those in Table 1.

**Table 3:** Thermal properties of native, phosphorylated and acetylated chestnut starch.

Samples	T_o_ (°C)	T_p_ (°C)	T_c_ (°C)	ΔH (J/g)
NS	57.4 ± 0.3a	65.6 ± 0.1b	75.1 ± 0.2a	9.6 ± 0.3a
PCS-1	45.3 ± 0.2c	54.1 ± 0.3d	61.1 ± 0.3d	4.6 ± 0.1d
PCS-2	45.1 ± 0.4c	49.7 ± 0.2e	58.7 ± 0.3e	3.7 ± 0.1e
PCS-3	44.1 ± 0.3d	48.4 ± 0.2f	56.1 ± 0.4f	2.9 ± 0.1f
ACS-1	57.5 ± 0.2a	65.0 ± 0.2c	74.6 ± 0.4a	9.5 ± 0.3a
ACS-2	55.9 ± 0.3b	66.4 ± 0.1a	73.9 ± 0.3b	6.9 ± 0.3b
ACS-3	56.2 ± 0.1b	66.1 ± 0.3a	72.7 ± 0.3c	5.9 ± 0.1c

Values are means ± SD. Values with the same letters in the same column are not significantly different (*p* < 0.05). Note: abbreviations are the same as those in Table 1.

**Table 4:** Pasting properties of native, phosphorylated and acetylated chestnut starch.

Samples	Peak Viscosity (cP)	Trough Viscosity (cP)	Breakdown Viscosity (cP)	Final Viscosity (cP)	Setback Viscosity (cP)	Pasting Temperature (°C)
NS	1796 ± 23.9d	1351 ± 25.7c	445 ± 18.4c	2260 ± 21.0c	909 ± 18.4c	74.2 ± 0.2a
PCS-1	3028 ± 32.4c	1378 ± 31.1c	1650 ± 28.3b	2117 ± 34.6d	739 ± 27.6de	50.2 ± 0.1c
PCS-2	3639 ± 27.9b	1978 ± 25.5b	1661 ± 24.0b	3121 ± 38.8b	1143 ± 14.0b	50.2 ± 0.2c
PCS-3	5532 ± 32.2a	2085 ± 23.9a	3447 ± 29.7a	3586 ± 30.9a	1501 ± 36.7a	50.2 ± 0.3c
ACS-1	1578 ± 35.4e	1301 ± 22.6cd	277 ± 9.2d	1995 ± 28.3e	694 ± 11.3e	71.9 ± 0.4b
ACS-2	1742 ± 25.5d	1329 ± 15.6cd	413 ± 17.7c	2123 ± 22.5d	794 ± 21.9d	71.9 ± 0.4b
ACS-3	1600 ± 19.8e	1283 ± 20.5d	317 ± 8.5d	2001 ± 18.2e	718 ± 19.1e	71.9 ± 0.3b

Values are means ± SD. Values with the same letters in the same column are not significantly different (*p* < 0.05). Note: abbreviations are the same as those in Table 1.

**Table 5:** Englyst digestion profiles of starch fractions from native, phosphorylated and acetylated chestnut starch (excluding free glucose, g/100 g).

Sample	RDS	SDS	RS
NS	8.2 ± 0.2f	33.6 ± 0.1a	58.2 ± 0.4e
PCS-1	14.5 ± 0.2c	9.3 ± 0.4c	76.2 ± 0.2b
PCS-2	22.5 ± 0.3b	13.1 ± 0.1b	64.4 ± 0.4c
PCS-3	24.0 ± 0.4a	13.2 ± 0.3b	62.7 ± 0.2d
ACS-1	12.8 ± 0.1d	4.9 ± 0.1d	82.3 ± 0.1a
ACS-2	11.3 ± 0.1e	4.7 ± 0.2d	84.0 ± 0.2a
ACS-3	14.0 ± 0.2c	4.6 ± 0.1d	81.4 ± 0.2a

Values are means ± SD. Values with the same letters in the same column are not significantly different (*p* < 0.05). Note: abbreviations are the same as those in Table 1.

## Data Availability

Not applicable.

## References

[1] Nep E.I., Ngwuluka N.C., Kemas C.U., Ochekpe N.A. (2016). Rheological and structural properties of modified starches from the young shoots of Borassus aethiopium. Food Hydrocoll..

[2] Rincón-Aguirre A., Pérez L.A.B., Mendoza S.A. (2018). Physicochemical Studies of Taro Starch Chemically Modified by Acetylation, Phosphorylation, and Succinylation. Starch/Starke.

[3] Kaur B., Fazilah A., Bhat R., Karim A.A. (2012). Progress in starch modification in the last decade. Food Hydrocoll..

[4] Miao M., Li R., Jiang B., Cui S.W., Zhang T., Jin Z. (2014). Structure and physicochemical properties of octenyl succinic esters of sugary maize soluble starch and waxy maize starch. Food Chem..

[5] Masina N., Choonara Y.E., Kumar P., Toit L.C.D., Govender M., Indermun S., Pillay V. (2017). A review of the chemical modification techniques of starch. Carbohyd. Polym..

[6] Singh J., Kaur L., McCarthy O.J. (2007). Factors influencing the physico-chemical, morphological, thermal and rheological properties of some chemically modified starches for food applications—A review. Food Hydrocoll..

[7] Dong H., Vasanthan T. (2020). Effect of phosphorylation techniques on structural, thermal, and pasting properties of pulse starches in comparison with corn starch. Food Hydrocoll..

[8] Leonel M., Bema M.S.D., Santos T.P.R.D., Franco C.M. (2021). L Preparation and properties of phosphate starches from tuberous roots. Int. J. Biol. Macromol..

[9] Yang L., Zhou Y., Wu Y., Meng X., Jiang Y., Zhang H., Wang H. (2016). Preparation and physicochemical properties of three types of modified glutinous rice starches. Carbohydr. Polym..

[10] Passauer L., Bender H., Fischer S. (2010). Synthesis and characterisation of starch phosphates. Carbohyd. Polym..

[11] Colussi R., Pinto V.Z., Halal S.L.M.E., Vanier N.L., Villanova F.A., Silva R.M.E., Zavareze E.D.R., Dias A.R.G. (2014). Structural, morphological, and physicochemical properties of acetylated high-, medium-, and low-amylose rice starches. Carbohydr. Polym..

[12] Hong J., Zeng X.A., Buckow R., Han Z., Wang M. (2016). Nanostructure, morphology and functionality of cassava starch after pulsed electric fields assisted acetylation. Food Hydrocoll..

[13] Singh J., Kaur L., Singh N. (2004). Effect of acetylation on some properties of corn and potato starches. Starch/Starke.

[14] Singh N., Chawla D., Singh J. (2004). Influence of acetic anhydride on physico-chemical, morphological and thermal properties of corn and potato starch. Food Chem..

[15] Włodarczyk-Stasiak M., Mazurek A., Kowalski R., Pankiewicz U., Jamroz J. (2017). Physicochemical properties of waxy corn starch after three-stage modification. Food Hydrocoll..

[16] Sodhi N.S., Singh N. (2005). Characteristics of acetylated starches prepared using starches separated from different rice cultivars. J. Food Eng..

[17] Guo J., Kong L., Du B., Xu B. (2019). Morphological and physicochemical characterization of starches isolated from chestnuts cultivated in different regions of China. Int. J. Biol. Macromol..

[18] De Vasconcelos M.C., Bennett R.N., Rosa E.A., Ferreira-Cardoso J.V. (2010). Composition of European chestnut (*Castanea sativa* Mill.) and association with health effects: Fresh and processed products. J. Sci. Food Agric..

[19] Wang M., Wu Y., Liu Y., Ouyang J. (2020). Effect of Ultrasonic and Microwave Dual-Treatment on the Physicochemical Properties of Chestnut Starch. Polymers.

[20] Liu C., Wang S., Chang X., Wang S. (2015). Structural and functional properties of starches from Chinese chestnuts. Food Hydrocoll..

[21] Zhang L., Liu T., Hu G., Guo K., Wei C. (2018). Comparison of Physicochemical Properties of Starches from Nine Chinese Chestnut Varieties. Molecules.

[22] Liu W., Wang R., Li J., Xiao W., Rong L., Yang J., Wen H., Xie J. (2021). Effects of different hydrocolloids on gelatinization and gels structure of chestnut starch. Food Hydrocoll..

[23] Liu T., Ma M., Guo K., Hu G., Zhang L., Wei C. (2019). Structural, thermal, and hydrolysis properties of large and small granules from C-type starches of four Chinese chestnut varieties. Int. J. Biol. Macromol..

[24] Wang S., Liu C., Wang S. (2016). Drying methods used in starch isolation change properties of C-type chestnut (*Castanea mollissima*) starches. LWT Food Sci. Technol..

[25] Hu N., Li L., Tang E., Tang E., Liu X. (2020). Structural, physicochemical, textural, and thermal properties of phosphorylated chestnut starches with different degrees of substitution. J. Food Process. Preserv..

[26] Wani I.A., Gani A., Sharma P., Wani T.A., Masoodi F.A., Hamdani A., Muzafar s. (2015). Effect of acetylation on the physico-chemical properties of Indian Horse Chestnut (*Aesculus indica* L.) starch. Starch/Starke.

[27] Chrastil J. (1987). Improved colorimetric determination of amylose in starches or flours. Carbohydr. Res..

[28] Konik-Rose C.M., Moss R., Rahman S., Appels R., Stoddard F., McMaster G. (2001). Evaluation of the 40 mg swelling test for measuring starch functionality. Starch/Starke.

[29] Liu C., Wang S., Wang S. (2015). Physicochemical properties and in vitro digestibility of starches from ield peas grown in China. LWT Food Sci. Technol..

[30] Englyst H.N., Kingman S.M., Cummings J.H. (1992). Classification and measurement of nutritionally important starch fractions. Eur. J. Clin. Nutr..

[31] Nathania I., Sugih A.K., Muljana H. (2017). Preliminary Study on the Synthesis of Phosphorylated Mung Bean Starch: The Effect of pH on the Physicochemical and Functional Properties. Indones. J. Chem..

[32] Code of Federal Regulations (1994). Food Additives Permitted in Food for Human Consumption.

[33] Siroha A.K., Sandhu K.S., Kaur M., Kaur V. (2019). Physicochemical, rheological, morphological and in vitro digestibility properties of pearl millet starch modified at varying levels of acetylation. Int. J. Biol. Macromol..

[34] Mbougueng P.D., Tenin D., Scher J., Tchiegang C. (2012). Influence of acetylationon physicochemical, functional and thermal properties of potato and cassava astarches. J. Food Eng..

[35] Rahim A., Kadir S., If’all, Asriani Hasanuddin (2020). Characteristics of Acetylated Banggai Yam Starch on pH and Concentration of Anhydride Acetic. Int. J. Adv. Sci. Technol..

[36] Rożnowski J., Przetaczek-Rożnowska I., Boba D. (2016). Physicochemical properties of native and phosphorylated pumpkin starch. Starch/Starke.

[37] Lawal M.V., Odeniyi M.A., Itiola O.A. (2015). Material and rheological properties of native, acetylated, and pregelatinized forms of corn, cassava, and sweet potato starches. Starch/Starke.

[38] Olagunju A., Omoba O.S., Enujiugha V.N., Wiens R.A., Gough K.M., Aluko R.E. (2020). Influence of acetylation on physicochemical and morphological characteristics of pigeon pea starch. Food Hydrocoll..

[39] Yadav D.K., Patki P.E. (2015). Effect of acetyl esterification on physicochemical properties of chick pea (*Cicer arietinum* L.) starch. J. Food Sci. Technol..

[40] Singh H., Sodhi N.S., Singh N. (2012). Structure and functional properties of acetylated sorghum starch. Int. J. Food Prop..

[41] Akhilesh V.S., Lila K.N. (2012). Synthesis and evaluation of physicochemical properties cross-linked sago starch. Int. J. Biol. Macromol..

[42] Chang Y., Lv Y. (2017). Structure, functionality, and digestibility of acetylated hulless barley starch. Int. J. Food Prop..

[43] Xu S.S., Zhang J.X., Li B., Li J., Zhou B., Yan J.J., Song R.K. (2012). Preparation and physical characteristics of resistant starch (type 4) in acetylated indica rice. Food Chem..

[44] Colussi R., El Halal S.L.M., Pinto V.Z., Bartz J., Gutkoski L.C., Zavareze E.R., Dias A.R.G. (2015). Acetylation of rice starch in an aqueous medium for use in food. LWT Food Sci. Technol..

[45] Sindhu R., Devi A., Khatkar B.S. (2021). Morphology, structure and functionality of acetylated, oxidized and heat moisture treated amaranth starches. Food Hydrocoll..

[46] Luo Z.G., Shi Y.C. (2012). Preparation of acetylated waxy, normal, and high-amylose maize starches with intermediate degrees of substitution in aqueous solution and their properties. J. Agric. Food Chem..

[47] Wojeicchowski J.P., Siqueira G.L.A., Lacerda L.G., Schnitzler E., Demiate I.M. (2018). Physicochemical, structural and thermal properties of oxidized, acetylated and dual-modified common bean (*Phaseolus vulgaris* L.) starch. Food Sci. Technol..

[48] Ayucitra A. (2012). Preparation and Characterisation of Acetylated Corn Starches. Int. J. Chem. Eng. Appl..

[49] Ramadan M.F., Sitohy M.Z. (2020). Phosphorylated Starches: Preparation, Properties, Functionality, and Techno-Applications. Starch/Starke.

